# Red blood cell vesiculation in hereditary hemolytic anemia

**DOI:** 10.3389/fphys.2013.00365

**Published:** 2013-12-13

**Authors:** Amr Alaarg, Raymond M. Schiffelers, Wouter W. van Solinge, Richard van Wijk

**Affiliations:** ^1^Department of Clinical Chemistry and Haematology, University Medical Center UtrechtUtrecht, Netherlands; ^2^Department of Pharmaceutical Sciences, Utrecht UniversityUtrecht, Netherlands

**Keywords:** microvesicle, red blood cell, hemolytic anemia, membrane disorder, enzyme disorder, hemoglobinopathy, erythrocyte

## Abstract

Hereditary hemolytic anemia encompasses a heterogeneous group of anemias characterized by decreased red blood cell survival because of inherited membrane, enzyme, or hemoglobin disorders. Affected red blood cells are more fragile, less deformable, and more susceptible to shear stress and oxidative damage, and show increased vesiculation. Red blood cells, as essentially all cells, constitutively release phospholipid extracellular vesicles *in vivo* and *in vitro* in a process known as vesiculation. These extracellular vesicles comprise a heterogeneous group of vesicles of different sizes and intracellular origins. They are described in literature as exosomes if they originate from multi-vesicular bodies, or as microvesicles when formed by a one-step budding process directly from the plasma membrane. Extracellular vesicles contain a multitude of bioactive molecules that are implicated in intercellular communication and in different biological and pathophysiological processes. Mature red blood cells release in principle only microvesicles. In hereditary hemolytic anemias, the underlying molecular defect affects and determines red blood cell vesiculation, resulting in shedding microvesicles of different compositions and concentrations. Despite extensive research into red blood cell biochemistry and physiology, little is known about red cell deformability and vesiculation in hereditary hemolytic anemias, and the associated pathophysiological role is incompletely assessed. In this review, we discuss recent progress in understanding extracellular vesicles biology, with focus on red blood cell vesiculation. Also, we review recent scientific findings on the molecular defects of hereditary hemolytic anemias, and their correlation with red blood cell deformability and vesiculation. Integrating bio-analytical findings on abnormalities of red blood cells and their microvesicles will be critical for a better understanding of the pathophysiology of hereditary hemolytic anemias.

## Introduction

Red blood cells (RBCs) are the most abundant cell type in human blood and they function to transport oxygen (O_2_) from the lung to all tissues and cells, and to transport carbon dioxide (CO_2_) from tissues back to the lung. These functions dictate the unusual capacity of RBCs to pass through all types of vessels, and even to squeeze in capillaries of smaller diameters than RBCs themselves (Mokken et al., 1992). Indeed, the molecular structures of normal RBC membrane, cellular content, and energy machinery enable such extraordinary deformability under the high shear forces of blood flow (Svetina, 2012). Many patients with hereditary (inherited) hemolytic anemias show aberrant RBC deformability due to defects in one or more of RBC molecular components which are crucial for the mechanical strength of RBCs as well as their protection from oxidative stress (Hebbel, 1991; Gurbuz et al., 2004; Da Costa et al., 2013). Non-immune hereditary hemolytic anemias may be classified according to the underlying defects into three major groups: membranopathies, enzymopathies, and hemoglobinopathies (Dhaliwal et al., 2004).

Hereditary spherocytosis (HS) is the most common type of hereditary hemolytic anemia. The estimated prevalence of this membrane disorder is 1 in 5000 in the white population of Northern Europe. Red blood cell glucose-6-phosphate-dehydrogenase (G6PD) deficiency is the most common enzyme disorder worldwide, affecting 420 million of the world population. Less common is hemolytic anemia due to pyruvate kinase (PK) deficiency with an estimated prevalence of 1 in 20,000 in the white population. Sickle cell anemia is the conspicuous example of anemia due to a hemoglobinopathy. Its prevalence is 1–5 in 10,000. For more prevalence data and other hereditary hemolytic anemias, the ENERCA (European NEtwork for Rare and Congenital Anaemias) website (www.enerca.org) and the portal for rare diseases and orphan drugs (www.orpha.net) are recommended.

Both in physiological and pathophysiological processes the role of extracellular vesicles is increasingly appreciated. Most cells, if not all, secrete extracellular vesicles, which comprise heterogeneous populations of vesicles of different compositions and physicochemical properties. Considerable evidence is accumulating showing the significance of extracellular vesicles as key players for intercellular communication, in health and disease (EL Andaloussi et al., 2013). Focusing on RBC vesiculation, both *in vivo* or under blood storage conditions *ex vivo* mature RBCs lose their membranes through shedding of microvesicles, a class of extracellular vesicles defined by the fact that they originate from the plasma membrane (Greenwalt, 2006). In hereditary hemolytic anemias, the molecular defects affect not only the RBC but also their normal vesiculation pattern, resulting in the release of circulating microvesicles which probably have a different composition compared to those derived from normal RBCs. Loss of RBC membrane as microvesicles likely alters the cell's surface area-to-volume (S/V) ratio and RBC internal viscosity, and hence, perturbs RBC deformability (Mohandas et al., 1980).

Alterations in RBC deformability can be measured using a laser diffraction technique known as ektacytometry. Using this technique, a thin layer of RBCs is sheared between two rotating surfaces, transforming RBCs from the discoid morphology into the elliptical one. The laser beam is deflected by RBCs to produce patterns from which RBC deformability is assessed (Mohandas et al., 1980). Ektacytometry is a robust and easy-to-perform technique, which can be routinely used to scan blood samples to provide valuable information about abnormalities of RBC deformability (Vent-Schmidt et al., 2013). Harnessing RBC deformability and the emerging findings in extracellular vesicle field may open up new avenues for understanding and diagnosing rare, possibly neglected, diseases like hereditary hemolytic anemias. This review provides brief insights into vesiculation, RBC-derived vesicles and RBC deformability while emphasizing their translational value for patients with hereditary hemolytic anemias.

### Extracellular vesicles and their pathophysiological significance

Intercellular communication was believed to occur only via cell-to-cell contact and/or secreted soluble factors. Within the last three decades, there has been a paradigm shift in studying extracellular vesicles as key mediators of intercellular communication. Extracellular vesicles are membranous lipid bilayer-vesicles ubiquitously secreted by different cell types. Although there are conserved vesicular components, the composition of extracellular vesicles considerably varies according to the secreting cells, the stimulus for their formation, in addition to the inter-individual variability (Thery et al., 2009; Bastos-Amador et al., 2012). Extracellular vesicles may be classified by their intracellular origins. For instance, a subtype of extracellular vesicles known as exosomes originate from multi-vesicular bodies, and they are secreted by a two-step process: inward budding of the plasma membrane to form multivesicular bodies (MVBs) followed by fusion of the MVBs with the plasma membrane. The second subtype of extracellular vesicles is known as microvesicles or ectosomes, which are released by outward budding from the plasma membrane (Thery et al., 2009).

Over the last two decades, extracellular vesicles have been intensively studied after finding that they are more than cellular artifacts or clearance machineries of cellular junk. Stegrnayr and Ronquist have published the first report on the functionality of extracellular vesicles, showing that prostasomes could promote human sperm motility (Stegmayr and Ronquist, 1982). However, the biological functions of extracellular vesicles remained underestimated and unanalyzed until 1996 when Raposo et al. reported that B lymphocytes-derived exosomes could stimulate adaptive immune responses (Raposo et al., 1996). This work stimulated the scientific community to investigate the biological functions of extracellular vesicles, especially that these vesicles are secreted by nearly all cell types, including stem cells, cancer cells, and cellular components of blood. Beside their ubiquitous secretion, extracellular vesicle formation and release probably occurs in a tightly controlled manner and their bioactive cargo is selectively sorted (Kriebardis et al., 2012).

Recently, advances in analytical techniques for nucleic acids and proteomics enabled the identification of mRNAs, regulatory miRNAs, and functional proteins loaded into/onto extracellular vesicles. Such findings emphasize the pathophysiological significance of extracellular vesicles, which may transfer these bioactive payloads from one cell to another cell in many diseases (Raposo and Stoorvogel, 2013). Pioneering work was performed by Valadi et al. who demonstrated that exosomes could transfer functional mRNA and miRNAs between cells (Valadi et al., 2007). Later, Skog et al. found that glioblastoma-derived microvesicles could promote tumor growth and angiogenesis via delivering angiogenic and invasiveness factors to recipient cells in the tumor microenvironment (Skog et al., 2008). Moreover, a growing body of findings shows the implications of extracellular vesicles that may fuel bacterial or viral virulence through transferring pathogenic factors, either in pathogen-derived vesicles or in vesicles released from pathogen hijacked-host cell vesiculation machineries (Silverman and Reiner, 2011). Very recent work shows the importance of circulating exosome-like vesicles released by *Plasmodium falciparum*-infected RBCs to the sexual stage of malarial infection (Regev-Rudzki et al., 2013).

### Red blood cell-derived vesicles

Red blood cells constitute 40% of the total blood volume and they are one of the major vesicle-secreting cells in the circulating blood (Xiong et al., 2012). Although the majority of circulating microvesicles in healthy individuals are derived from platelets, RBCs-derived microvesicles levels are elevated during the course of some pathological conditions such as malaria and sickle cell disease (Barteneva et al., 2013). Interestingly, extracellular vesicles were first discovered as released by blood cellular components. In 1967, extracellular vesicles were described by Wolf as products or dust that resulted from platelet membrane fragmentation in human plasma (Wolf, 1967). Later, in 1983, two independent groups reported the release of extracellular vesicles by exocytosis of multivesicular endosomes during the maturation of reticulocytes (Harding et al., 1983; Pan and Johnstone, 1983). Although reticulocytes, or immature RBCs, secrete exosomes during the remodeling process that accompanies their maturation, mature RBCs are probably the only cells that do not secrete exosomes and shed only microvesicles (de Vooght et al., 2013).

Red blood cell-derived microvesicles are submicron membranous structures with a lipid bilayer rich in different phospholipids and proteins, which are derived from parental RBCs. Their average size is approximately 100–200 nm.

During their 120-days life span, normal RBCs lose approximately 20% of their hemoglobin and membrane through vesiculation (Werre et al., 2004). Red blood cell vesiculation can be increased *in vitro* by increasing levels of intracellular calcium and by depleting endogenous ATP (Lutz et al., 1977; Bevers et al., 1992). Under blood storage conditions, RBCs undergo structural and morphological changes associated with ATP depletion and oxidative damage of membrane lipids and proteins. Such changes, known as storage lesions, eventually result in shedding microvesicles and affect RBC survival after blood transfusion (Bosman et al., 2008). These lesions are dependent on storage conditions, storage length, and additives used in blood products (Veale et al., 2011).

**Figure 1:**
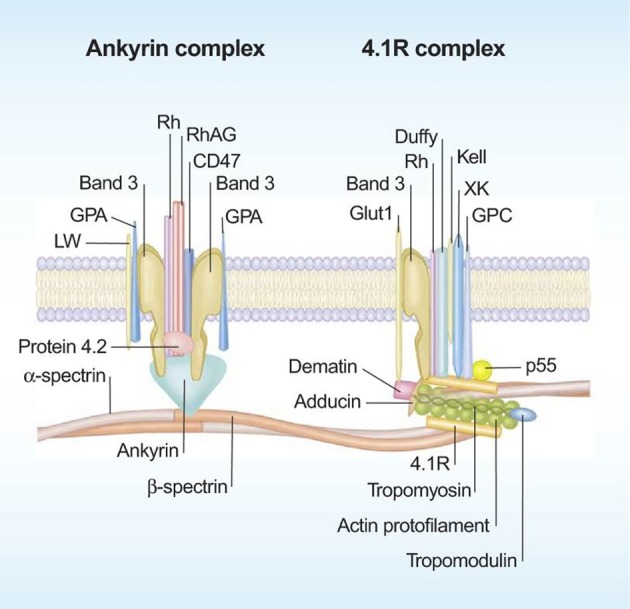
**Schematic representation of the red blood cell membrane**. The red blood cell membrane consists of a phospholipd bilayer that is anchored to the 2-dimensional elastic network of skeletal proteins (mainly spectrin) through transmembrane proteins (reproduced with permission from Mohandas and Gallagher, 2008).

Red blood cell membrane components are key players in microvesicle formation and release, and hence acknowledging their roles may improve our understanding for RBC vesiculation. The RBC membrane is composed of a phospholipid bilayer, which is penetrated by integral proteins (e.g., band 3, glycophorins A and C, Rhesus-associated antigen), and tethered to the cytoskeleton network composed of α- and β-Spectrin and actin via protein 4.1R, protein 4.2, and ankyrin (Figure 1). The RBC membrane and cytoskeleton proteins function to maintain the integrity of plasma membrane, and also to confer deformability and mechanical strength to RBCs (Mohandas and Gallagher, 2008). Besides, several RBC membrane proteins have transport functions. For instance, glucose transporter 1 (GLUT1) and aquaporin-1 controls transport of glucose and water, respectively. Also, multiple ion channels span the RBC membrane to regulate ion gradients and the hydration state of RBCs (Thomas et al., 2011).

**Figure 2:**
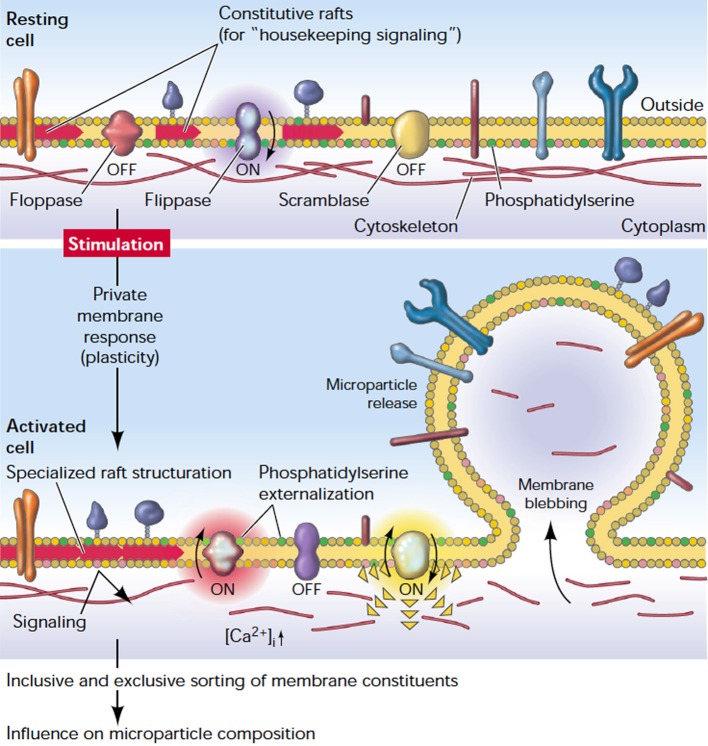
**The plasma membrane response to cell stimulation**. Depicted is a general plasma membrane: a well-structured entity characterized by a controlled transverse distribution of lipids and different kinds of (transmembrane) proteins, and laterally organized domains (rafts). Stimulation causes a redistribution of proteins, structuration of rafts, externalization of PS, and release of microparticles (reproduced with permission from Hugel et al., 2005).

In the resting state, RBC membrane phospholipids are asymmetrically distributed across the bilayer, meaning that the neutral choline-containing phospholipids, like phosphatidyl-choline and sphingomyelin, reside in the outer leaflet of the RBC while the charged amino-containing phospholipids, like phosphatidylserine (PS), phosphatidylinosirol, and phosphatidyl-ethanolamine, reside in the inner leaflet of the membrane (Zwaal and Schroit, 1997). Beside the RBC cytoskeleton, the asymmetry of phospholipids across the RBC membrane is crucial for maintaining RBC stability, deformability and mechanical strength (Manno et al., 2002). The asymmetric distribution of phospholipids is tightly controlled by three key enzymes. The first is an ATP-dependent flippase which pumps the amino-containing phospholipids from the outer leaflet to the inner leaflet. The second is an ATP-dependent floppase which controls the reverse transfer of the choline-containing phospholipids from the inner leaflet to the outer leaflet, at slower rate compared to flippases. The third enzyme is a divalent-cation activated scramblase which controls the bi-directional transfer of phospholipids down their concentration gradients to achieve a more symmetrical distribution of phospholipids (Graham, 2004). Disruption of the plasma membrane phospholipid asymmetry and externalization of PS are major steps in RBC vesicle formation and release (Graham, 2004) (Figure 2).

High levels of intracellular Calcium activate scramblase and inhibit flippase, and that results in a collapse of lipid asymmetry and externalization of PS. The role of the floppase enzyme on disturbing the membrane lipid asymmetry is not clear. However, it may be involved in the rapid translocation of PS to the external leaflet (Bevers et al., 1999; Graham, 2004). Meanwhile, the increase of intracellular calcium also activates proteolytic enzymes like calpain which breaks down the tethering points between the membrane cytoskeleton and the plasma membrane, facilitating the release of microvesicles (Morel et al., 2011). However, Knowles et al. reported that RBC cytoskeleton did not break down but retracted to the cell body during membrane deformation under shear stress (Knowles et al., 1997). This finding may explain the release of microvesicles, which are free from cytoskeletal components. A simulation study for RBC membrane demonstrated that the cell cytoskeleton may break and reform in a dynamic manner and this may confer solid-to-fluid transition characteristics to the RBC membrane under different stimuli (Li et al., 2007). It is noteworthy that both the calcium-induced phospholipid scrambling and the microvesicle release may occur in two independent pathways. Bucki et al. found that stabilizing the phospholipid asymmetry by spermine did not inhibit calcium-induced RBC vesiculation (Bucki et al., 1998). Yet the exact underlying molecular mechanisms of RBC vesiculation are far from being completely deciphered. It is not well known how different stimuli, like calcium influx, amphiphiles, shear stress, or ATP depletion, may induce shedding of microvesicles that have different compositions. Furthermore, the selective sorting of certain membrane components into microvesicles remains to be understood (Knowles et al., 1997). Although the blood storage remodeling process is presumed to be similar to RBC aging *in vivo*, the role of cytoskeleton proteins in vesicle formation may be different *in vitro* compared to *in vivo* (Bosman et al., 2010; Canellini et al., 2012). Moreover, RBCs of different phenotypes may shed microvesicles differently. For instance, although RBCs in Scott syndrome, a rare hereditary bleeding disorder, have a normal morphology and show no abnormalities in cytoskeleton composition, they resist vesiculation upon stimulation with the Calcium ionophore A23187 (Bevers et al., 1992). In a nutshell, RBCs derived microvesicles are not always equal, and both RBC phenotype and the triggering stimulus may determine the characteristics of the released microvesicles.

Several physiological and pathophysiological functions of RBC-derived microvesicles have been described. The main characteristic of RBCs-derived microvesicles is the externalization of the negatively charged PS, which has two main physiological implications: (1) Promoting coagulation; (2) Acting as an “eat me” signal for macrophages. The procoagulant activity of PS relies on the catalytic activity of PS. PS acts as a binding surface for prothrombinase and other coagulation factors, increasing the transformation rate of prothrombin into thrombin, a major coagulation molecule (Lentz, 2003). Although platelet-derived microvesicles play an essential role in haemostasis regulation, RBC-derived microvesicles could be equally important in certain pathological conditions like sickle cell disease (Tan and Lip, 2005; Weiss et al., 2011). Additionally, RBC-derived microvesicles may promote thrombus formation *in vivo* via exposing tissue factor, a key initiator of coagulation (Biro et al., 2003). Exposed PS is a proposed “eat-me” signal for phagocytes to engulf PS-exposing apoptotic cells. Such signaling provides a protection from the toxic effects of apoptotic cells (Chaurio et al., 2009). The same clearance mechanism may occur for PS-exposing microvesicles. It has been proposed that RBC vesiculation may serve as a self-protection mechanism to prevent the early removal of RBCs through removing the damaged components of the RBC membrane. Thus, RBC-derived microvesicles “sacrifice” themselves; the reticuloendothelial system removes the PS-exposing microvesicles instead of their parental RBCs, which probably have restored lipid asymmetry after vesiculation (Willekens et al., 2008).

Red blood cell-derived microvesicles may also be implicated in immunomodulation, nitric oxide (NO)–redox homeostasis, and in inflammatory reactions. These microvesicles may have immunosuppressive activities by down-regulating the macrophages that uptake them. Sadallah et al. showed that RBCs-derived microvesicles were taken up by macrophages and this inhibited the release of tumor necrosis factor-α (TNF-α) and interleukin 8 when these macrophage were exposed to zymosan A and lipopolysaccharide (LPS), well-recognized as macrophage stimulators (Sadallah et al., 2008). Such immune-suppressive activities may have implications in the post-transfusion immune-suppression and infections. On the other hand, Mantel et al. found that RBCs infected with *P. falciparum* could release microvesicles rich in host and parasites proteins and these microvesicles possessed a potent stimulatory effect on cells of the innate immune system (Mantel et al., 2013).

During blood storage, RBCs are subjected to storage lesions which results in reduced integrity of the RBC membrane, associated with releasing the RBC oxy-hemoglobin content as cell-free oxy-hemoglobin and vesicular oxy-hemoglobin. Both forms may have enhanced scavenging activities for NO, a potent vasodilator. Infusion of stored blood was shown to have a potent vasoconstrictive effect associated with the degree of storage-related hemolysis, potentially due to NO scavenging (Donadee et al., 2011). Regarding the potential pro-inflammatory activities of RBC-derived microvesicles, the vesicle membrane is a source for amino-containing phospholipids, which are substrates of the secretory phospholipase A2 (sPLA2) enzyme. Consumption of vesicular phospholipids by sPLA2 may results in production of lysophosphatidic acid, which is implicated in cell proliferation, migration and inflammatory reactions (Fourcade et al., 1995).

## Hereditary hemolytic anemia: understanding the molecular biology

Hereditary hemolytic anemias basically stem from intrinsic RBC disorders. In this review we focus on this group of disorders which can be due to mutations in genes that encode for: (1) a transmembrane/cytoskeleton protein; (2) an enzyme needed for a metabolic reaction in RBCs; or (3) hemoglobin. The phenotypes of hereditary hemolytic anemias range from being clinically asymptomatic to severe life threatening conditions. In this section we discuss the molecular bases of these disorders, and highlight their potential relationships with RBC vesiculation.

### Red blood cell membrane disorders

Red blood cell membrane proteins control the mechanical strength, the elastic deformability and the hydration state of RBCs. Defect in one or more of these proteins may lead to RBC membrane instability, increased membrane rigidity, and/or aberrant hydration state of RBCs. These perturbations often are associated with morphological changes and decreased life span of the RBCs.

#### Hereditary spherocytosis

Hereditary spherocytosis (HS) is the most common inherited hemolytic anemia due to a membrane defect. Inheritance is mostly autosomal dominant and peripheral blood smears of HS patients show spherocytes (sphere-shaped RBCs). However, spherocytic cells may also be seen in other acquired disorders like autoimmune hemolytic anemias, thermal injuries, and venom poisoning (Perrotta et al., 2008). The spherocytic morphology of RBCs is due to loss of membrane surface area relative to the cell volume. Most cases of HS are caused by deficiencies in the membrane or cytoskeleton proteins ankyrin, band 3, protein 4.2, β-spectrin, or α-spectrin. The clinical severity of HS varies according to the degree of spectrin loss, even if the primary defect is not in the gene encoding spectrin (Chasis et al., 1988; Eber et al., 1990). The aforementioned proteins are vital for the vertical tethering of the RBC cytoskeleton network with the membrane lipid bilayer, and their loss results in reduced mechanical strength and loss of membrane surface area through vesiculation. Deficiency of one or more of the cytoskeleton components may create an area of weakness in the membrane, facilitating RBC vesiculation. Alternatively, loss of one of the integral proteins, for instance band 3, may affect the membrane integrity. Thus, the released microvesicles may differ from those released due to deficiencies in cytoskeleton proteins. Indeed, in HS, RBC-derived microvesicles were found to have different compositions based on the underlying molecular defect. Reliene et al. showed that microvesicles released from spectrin/ankyrin-deficient RBCs were enriched in band 3 proteins. However, band 3-deficient RBCs did not lose their few band 3 proteins. Loss of band 3 in microvesicles released from spectrin/ankyrin-deficient RBCs reduced the binding of membrane-bound immunoglobulin G (IgG) to band 3 clusters on RBCs, and hence reduced opsonization and immune-mediated clearance of RBCs. In contrast, band 3-deficient RBCs did not lose their band 3 molecules, which remained attached to the cytoskeleton and clustered on the RBC membrane, and hence were opsonized by IgG *in vivo* (Reliene et al., 2002).

#### Hereditary elliptocytosis

Hereditary elliptocytosis (HE) is another diverse group of inherited RBC membrane disorders that are transmitted in an autosomal dominant manner with exception of pyropoikilocytosis (HPP), a severe form of HE, which is generally transmitted in an autosomal recessive manner. HE is characterized by elliptical RBCs (elliptocytes) on peripheral blood smears. However, elliptocytes may also be seen in thalassemia and megaloblastic anemias. The clinical manifestations of HE and HPP vary from silent asymptotic conditions to a severe life threatening hemolytic anemia (Nagel, 2006).

The hallmark of HE is the loss of mechanical stability of the RBC membrane due to defects in membrane proteins maintaining the lateral linkages of the RBC cytoskeleton. These defects include quantitative and/or qualitative defects in proteins α-spectrin, β-spectrin, and protein 4.1. The quantitative defects result from reduced expression of genes encoding for normal proteins. On the other hand, qualitative defects result in an alteration in the protein structure that affects the protein interaction with the adjacent proteins. HE RBCs are more susceptible to shear-stress induced fragmentation which is associated with membrane loss and progressive transformations of RBCs from the biconcave morphology to the elliptical or oval one. Membrane loss is potentially through shear-induced vesiculation, and such loss may be involved in decreased deformability of HE RBCs. However, the exact relationship between HE and vesiculation remains to be studied (Wagner et al., 1986; Gallagher, 2004).

#### Hereditary stomatocytosis

Hereditary stomatocytosis (HSt) is a genetic disorder that comprises a heterogeneous group of syndromes, in which the RBC membrane is leaky to monovalent cations, showing increased permeability to Na^+^ and K^+^. HSt is inherited in an autosomal dominant manner. HSt RBCs can be seen as mouth-shaped (stoma-) RBCs in peripheral blood smears. Like other RBC membrane disorders, the phenotype of HSt patients may range from being asymptomatic to suffering from a severe hemolytic anemia. Membrane leakiness of monovalent cations alters the osmotic pressure across the RBCs membrane, and hence affects RBC volume and cellular deformability. Such changes may decrease the life span of defective RBCs and increase their splenic sequestration (Bruce, 2009; Da Costa et al., 2013).

HSt can generally be classified into two main phenotypes: overhydrated HSt (OHSt) and dehydrated DHSt (also known as xerocytosis). In OHSt, RBCs have high intracellular sodium concentration, which results in cell overhydration, reduction in the mean cell hemoglobin concentration (MCHC), and transformation of RBCs into spherotic non-deformable cells. In xerocytosis, increased membrane leakiness, in particular K^+^, results in a reduction of total intracellular cation content. This induces RBC dehydration associated with elevated MCHC, and thus perturbs RBC deformability (Da Costa et al., 2013).

In OHSt, reduced levels of stomatin, a RBC membrane protein implicated in regulating K^+^/Na^+^ transport across the membrane, have previously been found. However, Stewart and Turner reported that stomatin knockout mice did not have hemolytic anemia, which may indicate that stomatin deficiency in OHSt is a secondary event and not the primary underlying molecular defect of DHSt (Stewart and Turner, 1999). In agreement with this, mutations in anion exchanger 1 (band 3) (Bruce et al., 2005), the putative ammonium transporter Rhesus-associated glycoprotein (RhAG) (Bruce, 2009), and GLUT1 (Flatt et al., 2011) have recently been implicated in OHSt. Some patients with HSt show normal levels of stomatin. It is worth mentioning that stomatin is a major lipid raft enriched in microvesicles derived from normal RBCs during blood storage. Actin-stomatin tethering may regulate the vertical association between the membrane and cytoskeleton, and hence regulate vesiculation. Indeed, Wilkinson et al. found that calcium-induced vesiculation is perturbed in stomatin-deficient RBCs of OHSt patients. By using calcium and the ionophore A23187, microvesicles-derived from stomatin-deficient RBCs were shown to be different in shape, size, number and composition when compared to microvesicles derived normal RBCs (Wilkinson et al., 2008). Yet, vesiculation of stomatin-deficient RBCs under physiological conditions remains to be studied. Some families affected by DHSt were recently shown to have mutations in PIEZO1. PIEZO proteins are pore forming subunits of mechanically activated cation channels (Andolfo et al., 2013). PIEZO1 mutations may be involved in leakiness of RBC cations through the membrane (Albuisson et al., 2013). It is not clear if RBC vesiculation is directly affected by mutations in PIEZO1 or if it occurs as a secondary event due to the perturbed deformability.

### Hereditary hemolytic anemia due to red blood cell enzyme disorders

Mature RBCs lack nuclei and mitochondria, and they have limited metabolic activities compared to other cells in the human body. Nevertheless, their limited metabolic pathways are capable of maintaining the RBC viability during its 120-days life span. These pathways are also crucial for O_2_ binding and delivery, through preserving hemoglobin in its functional form (Prchal and Gregg, 2005). Three main metabolic pathways exist in mature RBCs: (1) The anaerobic glycolysis pathway (Embden–Meyerhof pathway); (2) The pentose phosphate pathway; and (3) nucleotide metabolic pathways.

The Embden–Meyerhof pathway is the only source of energy in RBCs. Under physiological conditions, the majority of intracellular glucose is metabolized into pyruvate or lactate by the anaerobic Embden–Meyerhof pathway to generate Adenosine Triphosphate (ATP). The generated ATP is vital for RBC biological functions, including glycolysis itself, and cellular structures. For instance, ATP is essential for kinase reactions needed for cytoskeleton protein phosphorylation, which is crucial for the integrity and deformability of RBCs (Betz et al., 2009). Additionally, ATP molecules play a key role in membrane phospholipids asymmetry by controlling ATP-dependant phospholipids transporters (Daleke, 2008). In microcirculation, where O_2_ tension is low, RBCs release not only O_2_ but also ATP, whose vasodilating effects increase perfusion flow (Sprague and Ellsworth, 2012). In addition to generating ATP, Embden–Meyerhof pathway generates nicotinamide adenine dinucleotide (NADH), an essential molecule for reducing methemoglobin to hemoglobin. A shunt within the glycolysis pathway known as the Rapoport-Luebering shunt is responsible for the formation of 2,3-bisphosphoglycerate (2,3-BPG), an important cofactor for modulating hemoglobin affinity to O_2_for optimal O_2_ delivery to tissues (van Wijk and van Solinge, 2005).

The hexose monophosphate pathway consumes approximately 10% of the available glucose, under physiological conditions, to maintain a high ratio of NADPH to NADP. NADPH is critical to convert the oxidized form of glutathione (GSSG) to the reduced form (GSH) and thus protecting critical structures like membrane lipids and cytoskeleton proteins from oxidation. Under high oxidative stress conditions, the pentose phosphate pathway consumes more glucose to compensate for the depleted GSH (McMullin, 1999). Mature RBCs lack the ability for de novo synthesis of the adenine nucleotides needed for ATP production. Instead, salvage pathways are used to recycle the existing adenosine phosphate pool (Prchal and Gregg, 2005).

Multiple enzymes control the different metabolic pathways in RBCs, some of which are key enzymes in the concerning pathway. For example, glycolytic flux is mainly controlled by hexokinase, phosphofructokinase, glucose phosphate isomerase, and PK. The hexose monophosphate shunt is controlled by glucose-6-phosphate dehydrogenase. Pyrimidine 5′ nucleotidase is critically involved in the salvage pathway for nucleotide metabolism. Inherited abnormalities in one of these enzymes, hereditary enzymopathies, may result in chronic hemolytic anemias which vary in severity according to the relative importance of the mutated enzyme with regard to the functionality or the stability of this enzyme. The most prevalent hereditary enzymopathies of RBCs result from glucose-6-phosphate dehydrogenase (G6PD) deficiency and PK deficiency (van Wijk and van Solinge, 2005).

#### Glucose-6-phosphate dehydrogenase (G6PD) deficiency

G6PD deficiency is the most prevalent human enzyme deficiency, affecting hundreds of millions of people around the world. The disease is inherited as X-linked recessive trait. The majority of G6PD mutations are missense mutations in G6PD gene located on the X chromosome (band X q28). G6PD deficiency can be categorized based on the severity into three main variants: (1) Variants associated with episodic or acute hemolytic anemias; (2) Variants associated with chronic hemolytic anemias; (3) Variants associated with no clear risk of hemolysis (Gregg and Prchal, 2008).

G6PD activates the initial step of the hexose monophosphate shunt, which is needed to maintain high levels of NADPH and, consequently, GSH. Reduced glutathione is a sulfhydryl containing tripeptide acting intracellularly as a reducing agent. Reactive Oxygen species (ROS) are by-products of normal physiological intracellular reactions. However, ROS levels significantly rise upon exposure to exogenous factors like ingestion of drugs with high redox potentials, ingestion of fava beans, and during infections. Under normal conditions, oxidative damage is reversed by adequate levels of GSH. However, G6PD deficient RBCs have low levels of GSH, and they are more susceptible to damaging effects of the accumulated ROS resulting in hemolysis (Cappellini and Fiorelli, 2008). The exact molecular mechanisms of RBC hemolysis in G6PD deficiency are unclear. The intracellular accumulation of ROS may induce oxidation and clustering of membrane proteins, precipitation of oxidized hemoglobin on the inner membrane leaflet, and destabilization of the RBC membrane. Pantaleo et al. found an increase in oxidation and phosphorylation of band 3 proteins in G6PD-deficient RBCs, and that resulted in an increase in RBC hemolysis as well as an increase in the release of microvesicles containing hemichrome (Pantaleo et al., 2011).

Hemolytic episodes may stop despite the continuation of the drug administration or persistence of infection. This is probably due to the elimination of RBCs with lowest G6PD activity and, hence, lowest GSH levels. G6PD enzyme activity decreases during RBC aging; reticulocytes and younger RBCs usually have higher levels of G6PD. Red blood cell morphology may change in G6PD deficiency. Although in general RBC morphology is normal in RBC enzymopathies, peripheral blood smears of G6PD deficiency may display characteristic “bite cells” due to the oxidative denaturation of hemoglobin (Cappellini and Fiorelli, 2008).

#### Pyruvate kinase (PK) deficiency

Pyruvate kinase (PK) deficiency is the most common glycolytic enzyme defect and a relatively common cause of hereditary non-spherocytic hemolytic anemias. Patients have variable degrees of hemolysis, leading to mild compensated anemia, moderate hemolysis that exacerbates during infection, to severe transfusion-dependant chronic hemolytic anemia. The disease is due to missense mutations in the *PKLR* gene, resulting in generating a defective isoenzyme of PK-R, the PK isozyme which is specific to RBCs. PK deficiency is inherited in an autosomal recessive manner. PK is a key enzyme in Embden–Meyerhof glycolysis; it catalyses the transfer of a phosphoryl group from phosphoenolpyruvate to ADP to generate ATP (van Wijk and van Solinge, 2005). Although ATP molecules are crucial for the stability of the RBC membrane and for several enzymatic activities, the exact underlying mechanism of RBC hemolysis in PK deficiency is unclear. ATP deficiency is difficult to demonstrate, and other disorders accompanied with ATP depletion do not show hemolysis. Also, the selective splenic sequestration of young PK-deficient RBCs and its relationship with RBCs structure remain to be clarified (Zanella et al., 2005; Gregg and Prchal, 2008).

### Hereditary hemolytic anemias due to hemoglobinopathies

Hemoglobin is the main oxygen carrier in humans. It is a tetrameric protein composed of two pairs of different globin chains. Normal adult hemoglobin is mainly comprised of Hemoglobin A (Hb A), which is composed of two α-globin and two β-globin chains. Hemoglobin concentration (32–35 g per 100 mL of packed RBCs), the integrity and the solubility of these hemoglobin molecules, and their oxygen affinity are crucial factors for preserving the RBC's main function, oxygen delivery. More than 1000 mutations in globin-encoding genes have been found to affect hemoglobin synthesis, solubility, stability, or oxygen affinity. Such inherited hemoglobin anomalies may perturb the intracellular viscosity of RBCs and cell deformability, increasing the splenic sequestration of poorly deformable cells. Moreover, these perturbations may trigger proteolytic reactions, associated with ROS release, which may ultimately result in hemolytic anemias (Kuypers, 2008; Steinberg et al., 2008).

Hemoglobin anomalies can be associated with quantitative or qualitative abnormalities. Qualitative anomalies result from structurally altered hemoglobin, which has perturbed physical and/or chemical properties. Quantitative hemoglobinopathies are due to a defective production of otherwise normal globin chains. Examples of pathological conditions pertaining to the first group are sickle cell anemia, hereditary met-hemoglobinemia, high oxygen affinity polycythemia, and low oxygen affinity cyanosis. Examples of pathological conditions related to the quantitative anomalies are α- and β- thalassemias. By far the most prevalent and important conditions are sickle cell disease and the thalassemia (Poyart and Wajcman, 1996).

#### Sickle cell disease

Sickle cell disease is a hereditary disorder in which patients inherited a point mutation in the β-globin chain gene, encoding for valine instead of glutamic acid at position 6. With few exceptions, people with one sickle hemoglobin (HbS) gene are asymptomatic (sickle cell trait). People who have homozygosity for the mutation that causes HbS suffer from sickle cell disease. Sickle hemoglobin (HbS) polymerizes and undergoes transformation from the soluble state to a highly viscous semisolid gel under low oxygen tension conditions (Steinberg et al., 2008). An outcome of hemoglobin polymerization is the change in the morphology of RBCs, which *in vitro* become sickled, depending on the deoxygenation rate. HbS polymerization is different under *in vivo* conditions. The amount of polymer existing in the majority of cells *in vivo* is determined by the RBC hemoglobin content, the physiological oxygen tension, and the delay time for polymerization. Such factors affect the kinetics of polymerization and influence our understanding for the disease pathophysiology (Mozzarelli et al., 1987).

The clinical manifestations of sickle cell disease are downstream complications related to the formation of hyperdense RBCs (Lew and Bookchin, 2005) causing vaso occlusion. Main clinical symptoms include hemolytic anemia, recurrent painful episodes, chronic organ deterioration, particularly the spleen and kidneys, and various acute complications. However, the severity of sickle cell disease varies, and the genetics underlying this variability are far from being completely understood. One important phenotypic modifier concerns the co-inheritance of HbS with other mutant α- or β-thalassemia gene mutations (Poyart and Wajcman, 1996).

HbS polymerization cause structural damage, thereby altering the red cell membrane. In addition, unstable HbS may cause oxidative damage. Red blood cell membrane damages are associated with PS externalization and band 3 clustering, which may promote RBC opsonization, and consequently, RBC phagocytosis (Waugh et al., 1986; de Jong et al., 2001). Additionally, these membrane alterations may induce membrane surface area loss through releasing microvesicles. The release of microvesicles from sickled RBCs was first described by Allan et al. They reported that reoxygenation of sickled RBCs could result in the release of spectrin free- and polymerized hemoglobin-containing microvesicles (Allan et al., 1982). Others reported high levels of circulating microvesicles in patients with sickle cell anemia (Westerman et al., 2008).

#### Thalassemias

Thalassemias are a heterogeneous group of hereditary anemias which result from reduced biosynthesis of one of the two globin chains, α- or β-globin chain, that are needed to form the adult hemoglobin tetramer, HbA. Individual disorders of thalassemias are named according to the affected globin chain. For instance, patients with α-thalassemia have absent or reduced α-globin chains while patients with β-thalassemia have absent or reduced β-globin chains (Steinberg et al., 2008). The α- and β-globin chains in thalassemias are usually of normal structure.

Pathophysiological consequences of thalassemias arise from the underproduction of hemoglobin and the intracellular accumulation of excess globin subunits. The underproduction of hemoglobin diminishes oxygen carrying capacity of surviving RBCs. More importantly, the excess globin subunits probably precipitate on the membrane inner leaflet, inducing damages in the membrane cytoskeleton and decreasing the RBC deformability. Moreover, the rapid degradation of free globin chains in RBC precursors may result in destruction of RBC precursors in bone marrow associated with ineffective erythropoiesis (Poyart and Wajcman, 1996; Steinberg et al., 2008).

### The linkage between hereditary hemolytic anemias and red blood cell-derived microvesicles

The relationship between hereditary hemolytic anemias and RBC vesiculation is far from being completely deciphered. The diverse molecular pathologies of hereditary hemolytic anemias probably play a key role in determining RBC deformability, fragility and vesiculation. Thus, the phenotype and the levels of the circulating RBC-derived microvesicles of each disorder are primarily determined by the underlying molecular defects. Moreover, therapeutic interventions, like splenectomy and pharmaceutical compounds, may differently modify RBC vesiculation for each disorder (Bütikofer et al., 1989; Westerman et al., 2008).

Red blood cell deformability probably shows a negative correlation with RBC vesiculation. Such a correlation can be demonstrated by a reduction in the extent of RBC deformability, which is paralleled with an increase in microvesicle levels during blood storage (Almizraq et al., 2013). However, others found that there is a minimal change in RBC deformability during blood storage, and such a finding could be explained by the parallel decline in MCHC, which may compensate the effect of surface area loss on RBC deformability (Cluitmans et al., 2012). In hereditary membranopathies, like HS and HE, the instability of the RBC membrane is most likely accompanied by a consequent membrane loss through vesiculation, generating less deformable RBCs which have less surface/volume ratio. By using a quantitative flow cytometry method, Mullier et al. found that HS patients' blood contained higher levels of RBC-derived microvesicles compared to healthy controls (Mullier et al., 2012). Regarding hereditary hemoglobinopathies, unstable hemoglobin induces oxidation of membrane proteins and lipids, deposition of hemoglobin on the membrane, and changes in the intracellular viscosity, and that all results in poorly deformable RBCs (Mokken et al., 1992). Red blood cell tendency to shed microvesicles may reflect separation of the membrane bilayer from the underlying skeleton by spicules of polymerized deposited hemoglobin. For instance, in sickle cell disease, a substantial percentage of cell-free hemoglobin is encapsulated in RBC-derived microvesicles released during sickling. Concerning hereditary RBC enzymopathies, the correlation between RBC deformability and vesiculation is less clear than other hereditary hemolytic disorders, probably due to two main reasons; the genetic diversity of enzymopathies and the dependency of membrane damage on the level of oxidative stress. Johnson et al. reported increased deformability of G6PD-deficient RBCs, which may be explained by the fact that RBCs in many cases of glycolytic enzyme deficiency are foetal-like RBCs, which are more deformable (Johnson et al., 1999). However, others reported that G6PD-deficient RBCs were particularly susceptible to oxidative stress-induced damage, and that results in a dramatic reduction in RBC deformability (Gurbuz et al., 2004). Notably, it is likely that patients may have different subpopulations of RBCs, with different deformability, and that affects the overall deformability results (Mokken et al., 1992). The increased susceptibility of RBCs to oxidative damage in hereditary enzymopathies may cause microvesicle shedding. G6PD-deficient subjects may have high levels of circulating RBC- and platelet-derived microvesicles when compared to healthy controls, and microvesicle concentration probably has a negative correlation with G6PD activity (Nantakomol et al., 2012). However, vesiculation in other hereditary enzymopathies remains to be understood.

Treatment modalities of hemolytic anemia, like splenectomy and pharmaceutical compounds, may modify RBC deformability and vesiculation. The anatomic structure of the spleen is challenging for RBC metabolic machinery and deformability. RBCs have to go through repeated deformations when passing through both the capillary bed and the inter-endothelial slits of the spleen, where RBCs are concentrated and their intracellular metabolism is stressed. Patients with hereditary hemolytic anemias are likely to exhibit RBCs with morphological abnormalities or metabolically distressed RBCs, which cannot withstand such a splenic challenge. Thus, these abnormal RBCs are likely to be sequestered by the spleen and subsequently phagocytosed by splenic macrophages. Such splenic sequestration and erythrophagocytosis are responsible for the development of anemia and splenomegaly (Mebius and Kraal, 2005). Notably, in sickle cell disease the spleen is also one of the affected organs. During the first decade of life the spleen is usually enlarged but due to vaso occlusion events and infarction it undergoes progressive atrophy leading to autosplenectomy (Pearson et al., 1979, 1985; Al Salem, 2011).

Splenectomy is indicated as a last option to treat the progressive increase in blood transfusion requirements due to the splenomegaly-mediated hemolysis. In many cases of hereditary hemolytic anemias, splenectomy may improve anemia, reduce the rate of hemolysis, and eliminate symptoms of splenomegaly (Reliene et al., 2002; Casale and Perrotta, 2011). However, splenectomy is not a curative intervention, and sometimes contraindicated due to post-surgery complications. Although splenectomy may be beneficial for patients with HS or sickle cell disease, it is contra-indicated for patients with HSt, who may develop a hypercoagulable state after splenectomy resulting in thrombotic episodes or pulmonary hypertension (Stewart et al., 1996; Stewart and Turner, 1999). Also splenectomised β-thalassemia intermedia patients may develop venous thromboembolic complications like pulmonary embolism and deep venous thrombosis (Westerman et al., 2008). The underlying mechanisms of these thromboembolic events are not clear, and the role of RBCs-derived microvesicles in these events remains ill-defined. The transmembrane asymmetry of phospholipids and PS externalization may be different among the different hereditary hemolytic anemia, and these differences may play a crucial role in the hypercoagulable state. HS and HE RBCs were shown to have a normal transbilayer phospholipid asymmetry, whereas RBCs of some stomatocytosis variants were shown to have altered phospholipids asymmetry in addition to increased adherence to endothelial cells (de Jong et al., 1999; Gallagher et al., 2003). It is likely that splenectomy in stomatocytosis prolongs the circulation of the PS-exposing RBCs, developing a hypercoagulable state. Westerman et al. demonstrated that splenectomy may differently affect the levels of PS-exposing microvesicles between sickle cell anemia and thalassemia intermedia (Westerman et al., 2008). In patients with thalassaemia intermedia, splenectomy was shown to increase the levels of PS-exposing microvesicles, potentially due to the diminished clearance of abnormal RBCs, which produce these PS-exposing microvesicles. Although vesiculation may be facilitated by the spleen in healthy subjects, this would not explain the post-splenectomy increase in RBC-derived microvesicles in thalassaemia intermedia patients (Willekens et al., 2003; Westerman et al., 2008).

The use of medications for therapeutic needs should be carefully considered in patients with hereditary hemolytic anemias. Also, the diagnosis of such diseases and the analysis of RBCs-derived microvesicles in hemolytic anemias should be carefully performed with respect to the patient's medical history. Exposure of RBCs to pharmacologically active compounds, which have different physicochemical properties and mechanisms of actions, may have different impacts on RBC morphology, deformability and vesiculation. For instance, many drugs are amphiphilic in nature, meaning that that they have hydrophobic and polar parts.

Based on the bilayer couple hypothesis, which hypothesizes that RBC outer and inner leaflets respond differently to perturbations, the morphological transformations of RBCs, and the consecutive vesiculation, can be predicted from the nature of the drugs interacting with RBCs (Sheetz and Singer, 1974). For instance, anionic drugs, like indomethacine and phenyibutazone, preferentially accumulate in the RBC outer leaflet and induce RBC spiculation (echinocytosis) which results in membrane loss through vesiculation. Such an interaction is electrostatically more favorable due to the net negative charge of the inner membrane leaflet. On the other hand, cationic drugs, like chlorpromazine, primaquine and tetracaine, preferentially accumulate in the inner leaflet and they induce a stomatocytic transformation of RBC morphology, resulting in inhibition of vesiculation and/or endovesiculation (Bütikofer et al., 1987; Schreier et al., 2000). It is not clear if the physical expansion of the inner or the outer leaflet of the RBC membrane, based on the preferential accumulation of amphiphilic drugs, is the sole event that affects RBC transformations and vesiculation. Red blood cells possess membrane-associated enzymes, which have different topological arrangements/distribution across the cell membrane. For examples, ATPases, proteinases and acetylcholinesterase are known to be confined to the membrane, whereas enzymes of glutathione and glucose metabolism are found in the cytosol and associated with the inner leaflet of the membrane (Schrier, 1977). Some of these enzymes are involved in regulating RBC morphology, trafficking of cations, and cell membrane rigidity. In addition to the physical impacts, the intercalation of amphiphilic drugs may differently affect the membrane-associated enzymes based on which membrane leaflet is affected, and that may result in vectorial or one-sided changes in the membrane enzymatic activities, leading to differences in the RBC vesiculation behavior (Schrier, 1977; Bütikofer et al., 1989; Schreier et al., 2000). Additionally, the effects of amphiphilic drugs on RBC deformability and vesiculation can be explained by the possible mechanisms of action of antimicrobial peptides. An antimicrobial peptide, like gramicidin D, has amphiphilic nature, and it may affect the cell membrane integrity and ion permeability by two main possible mechanisms: (1) the carpet-like mechanism; and (2) the barrel-stave mechanism. In the first mechanism, the peptide acts as a detergent by lining on the cell membrane surface till reaching a critical concentration at which the lipid bilayer is disrupted. In the latter mechanism, a membrane channel is formed, in which the hydrophobic α- helical or β-sheets of the peptide interact with the acyl chains of the membrane whereas the hydrophilic portion lines the interior of the channel. Such a channel disrupts the osmotic state of the cell by disrupting membrane permeability (Schreier et al., 2000; Yeaman and Yount, 2003). Interestingly, beside amphiphilic drugs, other pharmaceutical molecules seem to intercalate with the RBC outer leaflet. For instance, Baerlocher et al. found that Fluorouracil (5-FU), an anti-cancer drug, could preferentially expand the outer leaflet of RBCs, inducing cell echinocytosis and shedding PS-exposing microvesicles. Such microvesicles are potent stimulators of coagulation and may be responsible for the thromboembolic complications that occur at high therapeutic doses of 5-FU (Baerlocher et al., 1997). However, others reported that the effects of 5-FU on RBCs may partially be explained by the depletion of RBC ATP (Spasojevic et al., 2005). Additionally, molecules that modulate calcium trafficking may be of a therapeutic value for conditions associated with RBCs of reduced deformability. Intracellular calcium plays crucial roles in controlling membrane deformability and vesiculation. An example of intracellular calcium modulators is zinc. Zinc therapy in sickle cell anemia may reduce RBC sickling and improve cellular deformability, potentially through inhibition of calmodulin, an intracellular mediator of Calcium activities (Brewer et al., 1977). Another example of a calcium antagonist is magnesium, which may be administered intravenously to reduce the risk of seizures in pregnant women with preeclampsia. Magnesium may partially improve the impaired microcirculation in preeclampsia by increasing RBC deformability through antagonizing the stimulation of calcium pumps and reducing ATP depletion (Schauf et al., 2004). An additional example is dipyridamole, an inhibitor of platelet activation, which may enhance RBC deformability by enhancing glucose uptake and boosting glycolysis, increasing intracellular levels of ATP, and thus maintaining a more efficient calcium pumping to the extracellular milieu (Sowemimo-Coker et al., 1983; Saniabadi et al., 1992).

### Integrated bio-analytical findings: a need for profiling red blood cells and red blood cell-derived microvesicles

Beside blood cell indices, current methodologies that are needed for the basic understanding and clinical diagnosis of hereditary hemolytic anemias encompass genetic analysis, protein analysis, assessment of RBC deformability, flow cytometry, assays to rule out immune-hemolytic anemia, and enzyme activity assays. Rarity and diversity of hereditary hemolytic anemias, and our incomplete understanding of RBC-derived microvesicles, which can be considered as miniatures of the aberrant RBCs, represent a challenge for the scientific community. Overcoming such a challenge is needed to have a better comprehension of the diseases and to develop new therapeutics for each disease. An approach to overcome these challenges is to combine and integrate data from the several experimental platforms on RBCs and their microvesicles to provide new insights into the molecular interactions, cellular and vesicular (sub) phenotypes, and disease processes. Such profiling of hereditary hemolytic anemias can be started by integrating data generated by robust methods like ektacytometry, flow cytometry, nanoparticle characterization and proteomics.

#### Cytometry

Ektacytometry is defined in the dictionary of cell and molecular biology as a “Method in which cells (usually RBCs) are exposed to increasing shear-stress and the laser diffraction pattern through the suspension is recorded; it goes from circular to elliptical as shear increases. From these measurements, a deformability index for the cells can be derived.” (Lackie, 2007). Determination of deformability index (DI), also known as elongation index (EI), is indicative to cell membrane rigidity. A variety of ektacytometry is the osmotic gradient ektacytometry, in which RBCs are suspended in solutions of polyvinylpyrrolidone (PVP) of varied tonicity and the DI is determined as a continuous function of the suspending medium osmolality at a constant shear rate. By keeping in mind that RBC membrane is highly permeable to water, any change in the osmolality of the suspension medium results in a change in the hydration state of RBCs (Johnson and Ravindranath, 1996). Red blood cell deformability is determined by RBC internal viscosity, membrane surface to volume ratio, and membrane rigidity. Alterations of one or more of these three determinants can be detected by interpreting changes in the osmotic gradient deformability profile. Since the underlying molecular defects of hereditary hemolytic anemias are different, a number of these anemias can be rapidly diagnosed by their distinctive osmotic gradient deformability profile (Clark et al., 1983; Johnson and Ravindranath, 1996; Da Costa et al., 2013). Combining these profiles with other haemorheological parameters like the aggregation behavior of RBCs may be of added value in further profiling of hereditary hemolytic anemias. An example of such a hybrid technique is the commercially available instrument called LORCA (Laser-assisted Optical Rotational Cell Analyzer) (Hardeman et al., 2001).

Flow cytometry is a simple, reproducible, and high throughput method that can be used for qualitative and quantitative immunophenotyping of RBCs. The availability of a wide array of fluorescently labeled antibodies can be utilized to detect several RBC surface and cytoplasmic antigens that may vary among hereditary hemolytic anemias. Additionally, laser scattering at different angles can distinguish differences in cell size and internal complexity (Brown and Wittwer, 2000; Davis, 2001). An automated hematology analyser like the Abbott cell-dyn Sapphire can also be modified to detect morphological abnormalities of the analyzed RBCs (Kim et al., 2003).

#### Nanoparticle characterization

The submicron size of microvesicles poses several challenges for analysis. To characterize their biological characteristics, such as surface proteins, tools that have originally been developed for cells are usually employed, like flow cytometry. The limited scattering properties of nanosized particles make flow cytometry of microvesicles not straightforward.

This is primarily caused by the lack of standardized assay protocols in addition to the heterogeneity in size (where the majority of the population usually is well below the detection threshold) and composition of microvesicles (Shah et al., 2008; Sustar et al., 2011). Another challenging factor is that the clearance of RBCs-microvesicles seems to be a very efficient process (Willekens et al., 2005). Thus, it is not clear if the flow cytometry quantification of these microvesicles represent their constant circulating levels *in vivo* or that these levels are highly variable in time. Thus, there is a need to standardize flow cytometry protocols and to profile the phenotypes of RBCs and their microvesicles in the different hemolytic anemias.

There are two main approaches that may be employed in analysing RBC-derived microvesicles: (1) selection of microvesicles based on a size criterion followed by analysis of the fluorescent signals of events limited to this preselected size, or (2) initial selection is based on the fluorescent signals then limiting the analysis by scatter gating to these signals (Xiong et al., 2012). Among the generally investigated antigens of RBCs and RBC-derived microvesicles are glycophorin A, PS, and band 3. Glycophorin A is expressed on the vast majority of RBC-derived microvesicles, and it is considered as a universal marker to distinguish RBC-derived microvesicles from those of other cell lineages (Pattanapanyasat et al., 2004). Vesicle PS can be labeled by Annexing V. However, it is not clear if all RBCs-derived microvesicles expose PS (Barteneva et al., 2013). Flow cytometry can also be used to quantify the transmembrane band 3 proteins by labeling with the dye eosin-5′ maleimide (EMA). EMA staining is frequently used in the diagnosis of HS (Girodon et al., 2008). As aforementioned, the molecular defects in HS are heterogeneous; a common feature of HS RBCs appears to be a weakening of the tethering points associated with band 3, and that facilitates RBC vesiculation and losing band 3 from the RBC membrane into the released microvesicle. Indeed, Stoya et al. and Knowles et al. reported that the EMA fluorescence signal was reduced in HS RBCs and after heat- or shear-induced vesiculation of normal RBCs, and the released microvesicles showed membrane associated-EMA labeled band 3 proteins (Knowles et al., 1997; Stoya et al., 1997). Although the EMA test is highly sensitive, it is not without drawbacks. EMA binds covalently to the Lys430 on the extracellular loop of band 3 protein but it may also bind to sulfhydryl groups expressed by Rh, RhAg and CD47. Moreover, some mutants of band 3 fail to bind to the dye EMA. The use of EMA test is also less effective in diagnosis of HS due to ankyrin defects (Girodon et al., 2008).

From the pharmaceutical technology field a number of techniques are available to characterize physicochemical properties of vesicles, such as size and surface charge. These can be measured by a variety of techniques such as dynamic light scattering, electron microscopy, tunable resistive pulse sensing, or nanoparticle tracking analysis. These techniques, however, have difficulties in assessing the biological heterogeneity of samples. As an example, nanoparticle tracking analysis studies the brownian motion of microvesicles, which can be tracked through the scattered light after laser illumination. Since the antigen number on the surface of nanosized vesicles is relatively small, it is difficult to extract biological information on surface markers as the absolute signal remains low.

#### The proteome of red blood cells and their microvesicles

Mature RBCs are anucleate cells with few intracellular structures and no capability to synthesize new proteins. Thus, their proteome is less complex compared to other cell types. This fact, along with the comprehension of RBC physiology, opens up an opportunity to use proteomic techniques for a better understanding of hereditary hemolytic anemias. Mass spectrometry (MS) is a powerful, automated and high throughput tool, which enables us to analyse the proteome of RBCs in health and disease. Such a comparative proteomic analysis can provide us with considerable insights into disease-related alterations and severity, potential diagnostic markers, potential therapeutic targets, and drug-induced changes (Prudent et al., 2011). Such studies may improve our understanding of the underlying complex pathophysiology of hereditary hemolytic anemias like sickle cell disease, HS and HE (Kakhniashvili et al., 2005; Margetis et al., 2007; Pasini et al., 2010; Yuditskaya et al., 2010). Despite advances in MS, proteomic studies of RBCs in hereditary hemolytic anemias are limited.

There is a growing interest in using proteomic technologies for high throughput protein profiling of extracellular vesicles. Such an interest is driven by new findings pointing to the crucial roles in intercellular communications and other pathophysiological processes. With respect to RBC-derived microvesicles, little is known about their protein cargo (Simpson et al., 2009). Proteomic data are mainly available on microvesicles derived from normal RBCs during storage under blood bank conditions (Tissot et al., 2013). Bosman et al., in a series of proteomic studies, investigated the effect of storage conditions and RBC ageing on RBC vesiculation (Bosman et al., 2008, 2010, 2012; Willekens et al., 2008). By comparing the proteome of RBC-derived microvesicles with the proteome of the parental RBC membrane, the Bosman group hypothesized that RBCs-derived microvesicles and vesiculation may be a means to selectively remove senescent cell antigens and other molecules to postpone the premature elimination of RBCs from the circulation. In the context of hereditary hemolytic anemias, Chaichompoo et al. showed that the levels of 29 proteins of microvesicles derived from β-thalassemia/hemoglobinE patients were significantly altered when compared to controls. These proteins included peroxiredoxin 6, apolipoprotein E, cyclophilin A and heat shock protein 90, and they were primarily involved in the regulation of RBC redox reactions, phospholipid turnover, and blood coagulation (Chaichompoo et al., 2012).

## Concluding remarks

Red blood cells-derived microvesicles, once thought of as cell debris, now seem to have important roles in regulating blood homeostasis and modulating immune response and other pathophysiological processes. Red blood cell microvesiculation is a tightly controlled process and sorting of bioactive molecules into microvesicles occurs in a selective manner. The molecular defects of hereditary hemolytic anemia are diverse, and they potentially affect RBC vesiculation. Microvesicles released in the different hereditary hemolytic anemias are potentially unequal and may have different biological effects. As the exact role of red cell derived miscrovesicles in many of the hemolytic anemias is currently unknown and unexplored this opens up a whole area of exciting research in the coming period of time. Understanding the molecular bases of hereditary hemolytic anemias, and their impact on RBCs deformability and microvesiculation, can provide new insights into the pathophysiology of these anemias and may lead to the discovery of new diagnostic markers and therapeutic molecules.

## Author contributions

Amr Alaarg, Raymond M. Schiffelers, Wouter W. van Solinge, and Richard van Wijk wrote the manuscript. All authors approved the final version of this manuscript.

### Conflict of interest statement

The authors declare that the research was conducted in the absence of any commercial or financial relationships that could be construed as a potential conflict of interest.
